# Preoperative High-Resolution Ultrasound-Guided Design of the Fibula Osteoseptocutaneous Flap: A Structured Surgeon-Performed Protocol

**DOI:** 10.3390/jcm15207768

**Published:** 2026-10-09

**Authors:** Alfredo Dente, Alberto Bolletta, Stefano Bacchini, Gaia Benvenuti, Anna Scarabosio, David Pakeliani, Eugenio Simoni, Eva Covello, Emanuele Cigna

**Affiliations:** 1 Plastic Surgery and Microsurgery Unit, Department of Translational Research and New Technologies in Medicine and Surgery, University of Pisa, 56126 Pisa, Italy; 2 Plastic and Reconstructive Surgery, Department of Medical Area (DMED), University Hospital Santa Maria della Misericordia of Udine, University of Udine, 33100 Udine, Italy; 3 Unit of Vascular Surgery, United Hospitals Villa Sofia-Cervello, 90146 Palermo, Italy

**Keywords:** fibula osteoseptocutaneous flap, CHFUScolor Doppler, flap design, computed tomographic angiography

## Abstract

**Background/Objectives:** The fibula osteoseptocutaneous flap is an established option for head and neck reconstruction. Preoperative planning requires assessment of donor-limb arterial anatomy and localization of cutaneous perforators. This study describes a structured surgeon-performed Conventional High-Frequency Ultrasound (CHFUS) protocol and evaluates its correlation with intraoperative findings. **Methods:** This retrospective study included patients undergoing reconstruction with a fibula osteoseptocutaneous free flap between January 2024 and December 2025. A standardized two-phase CHFUS workflow was used to assess infrapopliteal arterial anatomy and identify, characterize, and mark perforators at their deep fascial emergence. Preoperative findings were compared with intraoperative anatomy. **Results:** Ten patients underwent fibula flap reconstruction. Eighteen perforators were mapped, with a mean of 1.80 ± 0.63 per patient. The most common Kim–Lippert vascular pattern was type I (90%). Mean peroneal artery diameter at its origin was 2.83 ± 0.15 mm, and mean dominant perforator diameter at deep fascial emergence was 1.04 ± 0.14 mm. All 10 patients met the predefined anatomical agreement threshold of ≤1 cm (100%; exact two-sided 95% confidence interval, 69.2–100%). No flap required intraoperative redesign because of perforator anatomy. All flaps survived, without partial skin-paddle necrosis, donor-site ischemia, or vascular complications. **Conclusions:** This structured surgeon-performed CHFUS protocol was feasible and useful for fibula osteoseptocutaneous flap planning, enabling assessment of arterial anatomy, reliable perforator localization, and translation of preoperative findings into skin-paddle design. CHFUS should be considered complementary to CT angiography. Larger prospective studies are required to evaluate interoperator reproducibility and the diagnostic performance of CHFUS for clinically relevant arterial variants.

## 1. Introduction

The fibula osteoseptocutaneous free flap has become one of the most dependable composite flaps in reconstructive microsurgery because it combines a long segment of vascularized cortical bone, a reliable pedicle, the possibility of repeated osteotomies, and a cutaneous component suitable for simultaneous soft-tissue resurfacing [1,2,3]. Contemporary refinements in microsurgical technique, fixation, virtual surgical planning, and computer-aided design and manufacturing have further increased the precision of mandibular and maxillary reconstruction, but the biological success of the reconstruction still depends on vascular anatomy that must be understood before skin incision [4].

Preoperative donor-site assessment has two separate objectives. The first is vascular safety. Because the peroneal artery is sacrificed with the flap, clinically relevant infrapopliteal variants may make harvest unsafe despite apparently normal distal pulses. Dominant peroneal patterns, hypoplasia or aplasia of a tibial artery, and peronea arteria magna therefore require recognition before surgery [5]. The Kim–Lippert classification provides a practical framework for describing these branching variants and their surgical implications [6].

The second objective is flap design. The skin paddle depends on perforators whose number, caliber, course, and position vary between patients [7]. Septocutaneous perforators are usually favorable because they reach the skin through the posterior intermuscular septum, whereas musculocutaneous perforators may traverse soleus or, less commonly, flexor hallucis longus. Their precise emergence cannot be predicted reliably from surface landmarks alone, yet it influences skin-paddle orientation, the sequence of flap elevation, and the relationship between the soft-tissue component, fibular osteotomies, and pedicle.

Several imaging modalities are available. Handheld Doppler is inexpensive and convenient but provides an acoustic signal rather than a complete anatomical map and does not reliably define caliber, intramuscular course, or fascial penetration [8]. CTA provides a panoramic representation of lower-limb arterial anatomy and remains widely used when donor-limb vascular safety is the main question [9,10]. However, it is static, requires iodinated contrast and ionizing radiation, and does not reproduce the dynamic relationship between the mobile soft-tissue envelope and the operative position. MRA avoids radiation but is less routinely available.

Conventional high-frequency ultrasound (CHFUS), including color-coded duplex sonography, provides different and complementary information. It combines real-time anatomical visualization with flow assessment, can be performed by the microsurgeon, can be repeated immediately before surgery, and allows direct translation of imaging findings into skin markings [8,10,11,12,13]. Higher-frequency probes improve visualization of superficial small-caliber vessels, and structured surgeon-performed protocols have been described for several perforator donor sites [11,12,13,14,15,16,17]. Comparative lower-extremity studies have also shown high localization accuracy for color Doppler ultrasound relative to CTA [18,19].

The fibula flap is particularly suited to a combined ultrasound strategy because planning requires information at two very different depths. A lower-frequency examination can be used to identify the main infrapopliteal arterial axes and major branching variants, whereas a higher-frequency examination can then focus on the perforator network and its relationship with the posterior intermuscular septum and the proposed skin paddle. The most surgically useful target is not merely the presence of a perforator but its deep fascial emergence, because this point can be marked on the patient and directly encountered during flap elevation. A workflow based on this concept may reduce uncertainty at incision, facilitate deliberate perforator selection, and make the preoperative map more relevant to the actual dissection.

Despite the increasing use of surgeon-performed ultrasound in reconstructive microsurgery, dedicated CHFUS protocols for the osteoseptocutaneous fibula flap remain incompletely defined. The aim of this study was therefore to describe a standardized two-phase, surgeon-performed CHFUS protocol for preoperative planning of the fibula osteoseptocutaneous flap and to evaluate the agreement between preoperative ultrasound findings and intraoperative anatomy. The protocol was developed as a pragmatic operative tool rather than as a purely diagnostic imaging exercise: its purpose was to identify the relevant arterial pattern, characterize candidate perforators, mark the fascial emergence of the selected vessel, and translate these findings into the final flap design.

## 2. Materials and Methods

A retrospective study was performed at the Department of Plastic Surgery and Microsurgery, University Hospital of Pisa, Italy, including consecutive patients who underwent head and neck reconstruction with an osteoseptocutaneous fibula free flap between January 2024 and December 2025, after ablative oncological surgery. All included patients had a composite defect involving the mandible or maxilla and required a cutaneous component for soft-tissue reconstruction or monitoring.

Exclusion criteria were inoperable or metastatic oncological disease, severe systemic conditions precluding prolonged surgery, coagulation disorders, uncontrolled diabetes mellitus, significant peripheral vascular disease, previous major trauma or surgery involving either lower extremity, previous lower-limb radiotherapy, or ongoing chemotherapy or treatment with vasculotoxic agents considered potentially relevant to donor-vessel integrity. During the study period, CTA of the lower limbs formed part of the vascular assessment before fibula flap harvest. Patients in whom CTA demonstrated a vascular configuration considered unsafe for peroneal artery sacrifice, including a dominant peroneal artery pattern, peronea arteria magna, severe multifocal arterial calcification, or another abnormality potentially compromising distal limb perfusion, were likewise excluded from flap harvest. When the initially preferred donor limb was considered unsuitable, the contralateral lower extremity was assessed.

Before ultrasound mapping, each donor limb underwent a conventional clinical examination. Distal pulses were palpated, and the skin was inspected for trophic changes, scars, previous trauma, signs of venous or arterial disease, and factors that could interfere with flap elevation or donor-site closure.

During the first step, the fibular head, lateral malleolus, and posterior border of the fibula were identified by palpation and marked. A longitudinal fibular axis was drawn between the fibular head and lateral malleolus, and the projected posterior intermuscular septum was estimated from surface anatomy. These markings provided a reproducible reference frame for the subsequent ultrasound examination but were not used as a substitute for vascular mapping.

All CHFUS examinations were performed by the same reconstructive surgeon using a Resona 7 system (Shenzhen Mindray Bio-Medical Electronics Co., Shenzhen, China) with B-mode, color Doppler, and a 22 MHz linear-array transducer. Standardized presets were used. B-mode scanning employed a 10–20 MHz range, gain of 26–34 dB, depth of 2.2–2.9 cm, and focus at 1–2 cm. For color-flow assessment, the wall filter was minimized, gain was generally 8–18 dB, superficial-vessel frequency was 12–15 MHz, and velocity scale was approximately 4–9.5 cm/s to improve detection of low-flow perforators. Minor adjustments were allowed according to tissue depth. The ultrasound operator did not review the preoperative CTA before CHFUS mapping and was therefore blinded to CTA findings during the examination (Table 1).

The second step comprised the ultrasonographic vascular assessment and focused on donor-limb arterial anatomy. The ultrasound examination was performed with the patient supine, the knee slightly flexed, and the limb mildly internally rotated, reproducing the harvest position. The first phase assessed donor-limb arterial anatomy. Transverse scanning in the popliteal region identified the popliteal vessels, after which the probe advanced distally to the tibioperoneal trunk. The lower end of the available frequency range was used for deeper structures. The bifurcation into the posterior tibial and peroneal arteries was identified, and the peroneal artery was followed distally along the medial fibula with transverse and longitudinal scanning. Vessel continuity, caliber, flow, and the relationship between the infrapopliteal arterial axes were evaluated sufficiently to classify the branching pattern according to Kim–Lippert [6] and to recognize configurations suggesting peroneal dominance. This phase was intended to define the main anatomy relevant to harvest and to correlate it with the pre-existing CTA rather than reproduce a complete angiographic examination. CHFUS mapping was completed before the operator reviewed the CTA; comparison with CTA was performed subsequently as part of routine surgical planning.

After the main arterial axes had been defined, the examination transitioned to the second, higher-resolution phase dedicated to perforator mapping. The operating frequency was increased toward the higher end of the transducer range to improve spatial resolution. Color Doppler was used with low-flow settings to identify branches arising from the peroneal vascular system, with systematic scanning concentrated along the middle and distal thirds of the fibula, where clinically relevant septocutaneous perforators are frequently encountered. Candidate perforators were not accepted on the basis of a single color signal. Each vessel was dynamically followed from the peroneal system toward the skin, alternating longitudinal and transverse probe orientations to confirm continuity and to characterize its route through the deep tissues.

For each perforator, caliber, anatomical course, deep fascial penetration, and relationship to the posterior intermuscular septum and planned skin paddle were recorded. Septocutaneous and musculocutaneous courses were differentiated when possible. Septocutaneous vessels were followed through the posterior intermuscular septum, whereas musculocutaneous branches were traced through soleus or, less commonly, flexor hallucis longus. Relevant adjacent venous or neurovascular structures were also noted. Perforator diameter was measured on B-mode imaging in a true transverse plane at the level of deep fascial emergence. Color Doppler was used to confirm flow and vessel continuity but was not used to define the diameter measurement. During caliber assessment, an adequate layer of coupling gel and minimal transducer pressure were used to avoid deformation or collapse of the small-caliber vessel, and the vessel was centered in transverse section before caliper placement. This approach was used to minimize compression artifact and color-blooming-related overestimation. Diameter measurements were interpreted primarily as comparative values within the same device and preset because Doppler settings can influence apparent small-vessel caliber [12].

The point at which each perforator crossed the deep fascia was used as the principal surgical landmark. Once this point was identified with confidence, it was marked directly on the skin using an indelible marker. The perforator diameter was measured at this level, and its trajectory toward the peroneal system was reviewed before final selection. When more than one suitable perforator was present, the dominant perforator was chosen according to a combination of caliber, course, technical accessibility, relationship to the required skin paddle, and the anticipated complexity of dissection. A favorable septocutaneous course was preferred when it satisfied reconstructive requirements, but the protocol did not exclude musculocutaneous perforators when they were the most appropriate vessel for the planned flap.

**Figure 1:**
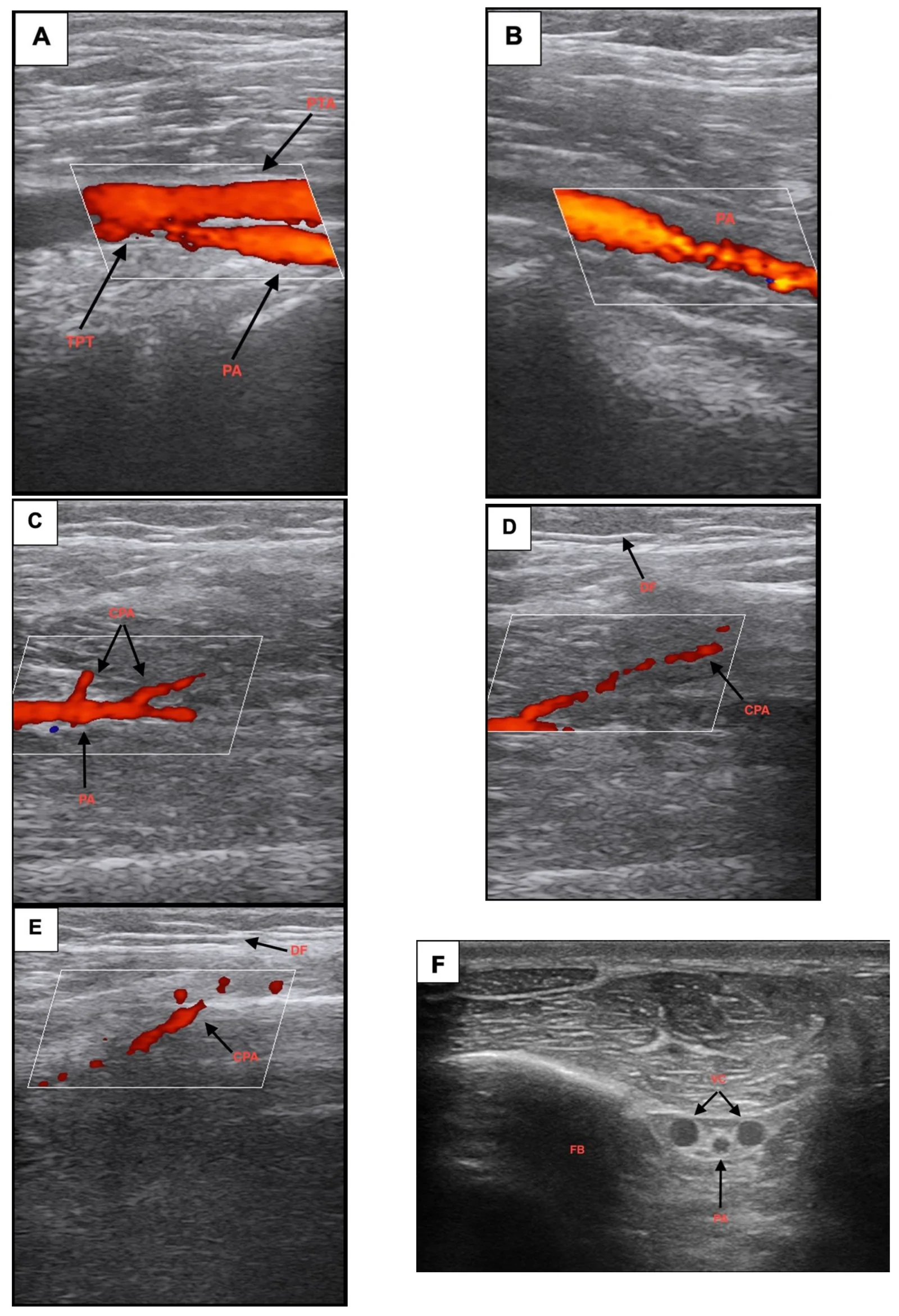

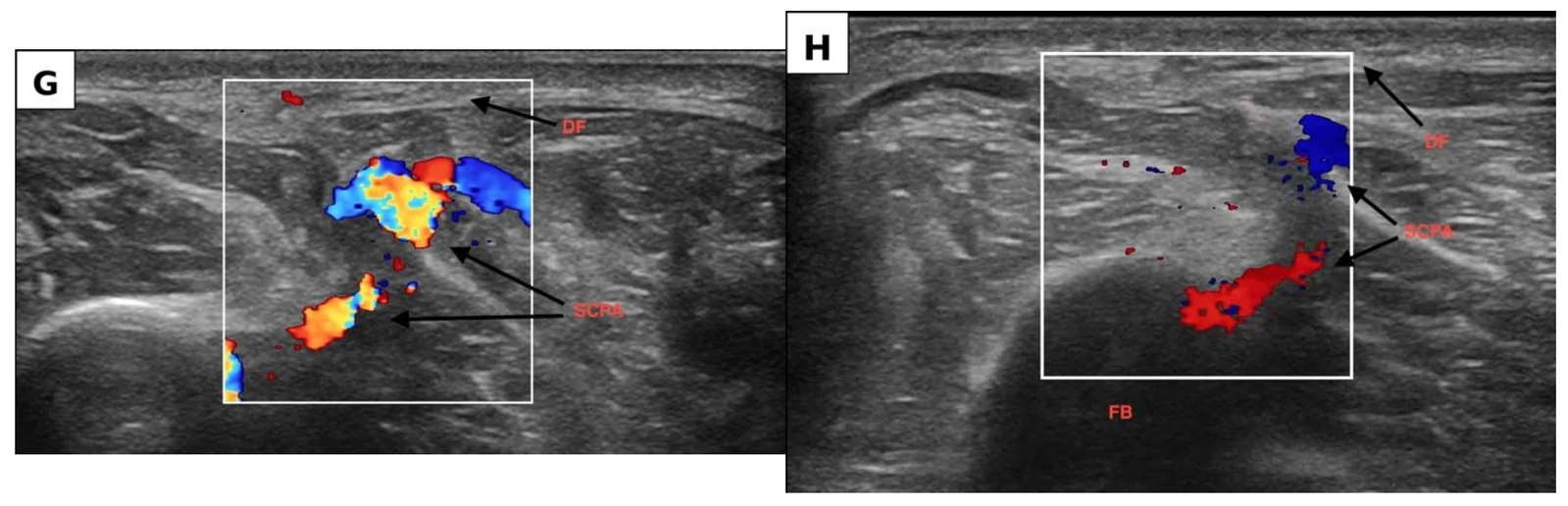
Preoperative ultrasound mapping of the osteoseptocutaneous fibula flap, progressing from assessment of the infrapopliteal vascular axis to detailed characterization of the cutaneous perforators. (**A**) Identification of the tibioperoneal bifurcation, with visualization of the posterior tibial and peroneal arteries using color Doppler imaging. (**B**) Longitudinal identification of the proximal peroneal artery after its origin from the tibioperoneal trunk. (**C**) Identification of a perforating branch arising from the peroneal vascular system and directed toward the superficial tissues. (**D**) Longitudinal tracking of a peroneal perforator from its source vessel toward the deep fascia. (**E**) Further visualization of the perforator along its course toward the superficial compartment. (**F**) High-frequency transverse B-mode image of the peroneal vascular bundle adjacent to the fibula, with the peroneal artery located between its accompanying veins. (**G**) Identification of a cutaneous perforator arising from the peroneal vascular system and coursing toward the deep fascia. (**H**) High-resolution visualization of the perforator at its deep fascial emergence, which represents the principal anatomical landmark used for preoperative skin marking and flap design. TPT, tibioperoneal trunk; PTA, posterior tibial artery; PA, peroneal artery; CPA, cutanepus perforating artery; DF, deep fascia; FB, fibular bone; VC, venae comitantes, SCPA, septocutaneous perforating artery.

**Figure 2:**
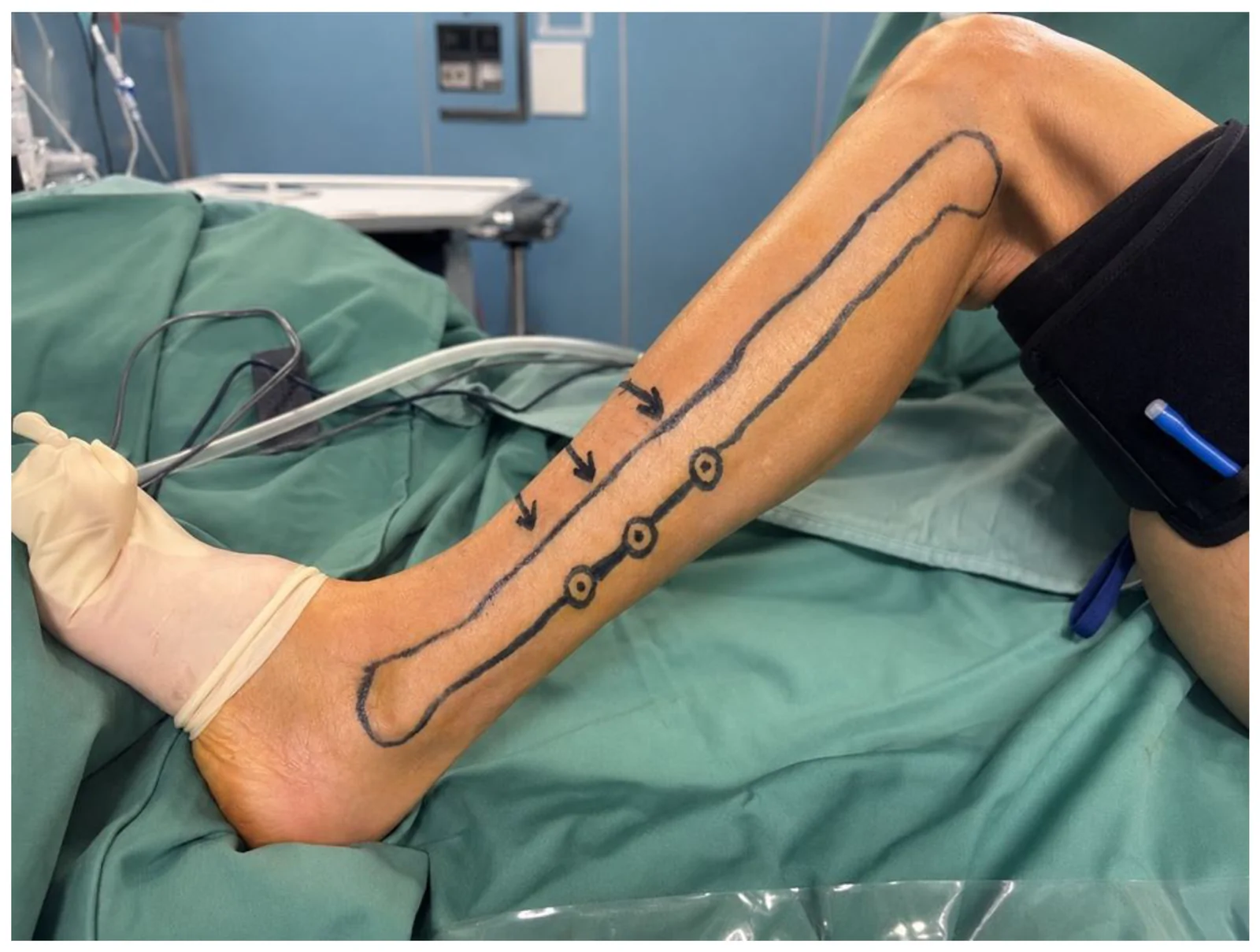
Preoperative CHFUS -guided design of the fibula osteoseptocutaneous flap. The fibular axis, planned skin paddle, and cutaneous perforator emergence points identified by ultrasound are marked directly on the donor limb.

The skin paddle was designed around the selected perforator and centered with reference to the posterior intermuscular septum, required defect dimensions, and planned fibular segment. The final markings included the fibular axis, estimated septal line, perforator emergence point or points, and skin-paddle orientation. When multiple perforators were identified, the design was adapted to the most favorable vessel while preserving the required relationship between cutaneous and osseous components. Representative arterial and perforator mapping and the resulting design are shown in Figure 1A–H, Figure 2, and Video S1.

The preoperative ultrasound map was systematically compared with intraoperative anatomy during flap elevation. Intraoperative anatomical correlation was assessed for the dominant perforator selected preoperatively for skin-paddle design. Its predicted fascial emergence point was compared with the corresponding intraoperative finding. Additional perforators identified during preoperative CHFUS mapping but not selected as the basis of the flap were not systematically assessed intraoperatively, and correlation data for these vessels were not collected. Anatomical agreement was defined as a distance of ≤1 cm between the preoperative skin marking of the selected dominant perforator and its corresponding intraoperative deep fascial emergence point. A distance >1 cm was classified as absence of agreement. Patient-level distances were reported for the selected dominant perforator and summarized as the median and range. The distance was measured in millimetres using a sterile soft ruler. The intraoperative localization measurement was performed by a reconstructive surgeon other than the surgeon who had conducted the preoperative CHFUS examination. The study specifically recorded whether the skin-paddle design required intraoperative modification because the perforator anatomy differed from the ultrasound map.

Primary outcome measures were intraoperative anatomical correlation, feasibility of ultrasound-guided flap design, number of perforators identified, peroneal artery diameter at its origin from the tibioperoneal trunk, dominant perforator diameter at deep fascial emergence, perforator distance from the fibular head, and infrapopliteal vascular pattern according to the Kim–Lippert classification. Operative variables included skin-paddle dimensions, number of perforators incorporated into the flap, recipient artery, and anatomical inset site. Secondary outcomes included flap- and donor-site complications, postoperative vascular complications, and the need for revision or flap redesign. Total flap loss and partial skin-paddle necrosis were classified as major flap complications; other events were classified as minor complications.

Statistical analysis was descriptive. Continuous variables were summarized as means and standard deviations, and categorical variables as counts and percentages. The proportion of patients meeting the predefined anatomical agreement threshold of ≤1 cm was reported with an exact two-sided 95% confidence interval calculated using the Clopper–Pearson method. The distance between the preoperative marking and the intraoperative deep fascial emergence point was additionally summarized as the median and range (minimum–maximum).

The study was conducted in accordance with the principles of the Declaration of Helsinki. Written informed consent was obtained from all patients for surgical treatment, ultrasound evaluation, and the use of anonymized clinical data and photographs for scientific purposes.

## 3. Results

Ten patients were evaluated during the study period, and all underwent preoperative CHFUS and CT angiography of the lower limbs. All 10 patients were included in the study, with no exclusions for any reason, including vascular abnormalities identified on CT angiography. The initially selected donor limb was used in every case; no patient required selection of the contralateral limb because of inadequate perforators in the initially selected limb.

The cohort included 6 men and 4 women, with a mean age of 52.10 ± 16.69 years and a mean body mass index of 22.98 ± 2.64 kg/m^2^. Seven patients had mandibular composite defects and three had maxillary defects. The mean postoperative follow-up was 12.9 months (range, 9–18 months). Patient-level demographic and defect characteristics are reported in Table 2.

Preoperative CHFUS was technically completed in every patient. The lower-frequency phase provided a continuous assessment of the relevant infrapopliteal vessels and allowed classification of arterial anatomy before perforator mapping. According to the Kim–Lippert classification used in the study, type I anatomy was identified in 9 patients (90%) and type IIA anatomy in 1 patient (10%). No type IIB or type III patterns were recorded in the included cohort, and no patient demonstrated a vascular configuration considered unsafe for fibula flap harvest. In particular, no case of peronea arteria magna was identified among the patients proceeding to surgery.

A total of 18 cutaneous perforators were mapped preoperatively, corresponding to a mean of 1.80 ± 0.63 perforators per patient; of the 18 mapped perforators, 15 (83.3%) were septocutaneous and 3 (16.7%) were musculocutaneous. Among the 10 dominant perforators selected for flap design, 9 (90%) were septocutaneous and 1 (10%) was musculocutaneous. Three patients had one mapped perforator, six had two, and one had three. Mean diameter of the peroneal artery at its origin from the tibioperoneal trunk was 2.83 ± 0.15 mm. The mean diameter of the dominant perforator at deep fascial emergence was 1.04 ± 0.14 mm. The mean distance between the fibular head and the first mapped perforator was 11.89 ± 0.98 cm. Among patients in whom a second perforator was identified, its mean distance from the fibular head was 19.49 ± 1.89 cm. A third perforator was present in one patient at 22.4 cm from the fibular head. Detailed patient-level ultrasound findings are summarized in Table 3.

The planned dominant perforator was identified intraoperatively in all 10 patients. The mean distance between the preoperative ultrasound skin marking and the corresponding intraoperative deep fascial emergence point was 5.28 ± 2.71 mm, with a median of 5.20 mm and a range of 1.20–9.70 mm. All patients met the predefined anatomical agreement threshold of ≤1 cm (10/10; 100%; exact two-sided 95% confidence interval, 69.2–100%). Patient-level localization distances and intraoperative characteristics are reported in Table 4.

The number, location, and general type of perforators identified on CHFUS were concordant with the anatomy encountered at flap elevation, and the preoperatively selected vessel could be used as the reference for skin-paddle design in all patients. No flap required intraoperative redesign because of an unexpected absence, displacement, or unfavorable course of the selected perforator.

**Figure 3:**
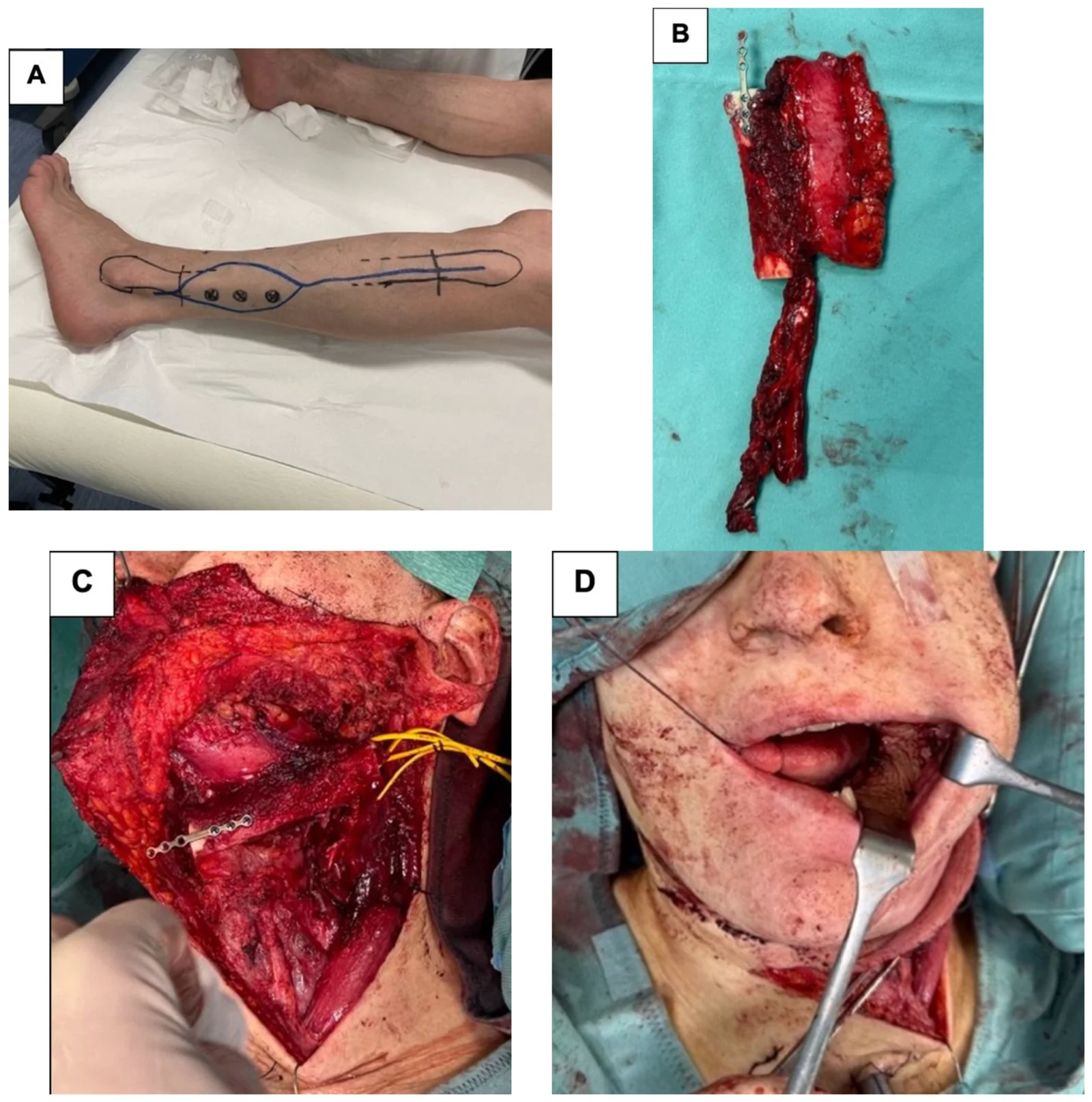
(**A**) Preoperative ultrasound-guided flap planning, with the fibular axis marked between the fibular head and the lateral malleolus and the cutaneous emergence points of three perforators identified and marked on the skin. (**B**) Harvested osteoseptocutaneous fibula free flap, including the osseous segment, skin paddle, and vascular pedicle. (**C**) Intraoperative inset of the vascularized fibular segment into the mandibular defect following segmental reconstruction. (**D**) Completed reconstruction, demonstrating restoration of mandibular continuity and resurfacing of the associated intraoral soft-tissue defect with the cutaneous paddle.

Although more than one perforator was mapped in several patients, one dominant perforator was incorporated into the skin paddle in every case. Skin-paddle dimensions ranged from 6 × 6 cm to 8 × 8 cm. The superior thyroid artery was used as the recipient artery in 8 patients (80%), while the facial artery was used in 2 patients (20%); both facial-artery recipient cases were maxillary reconstructions approached through the internal malar surface. Flap inset was performed for mandibular reconstruction in 7 patients and maxillary reconstruction in 3. The intraoperative characteristics are reported in Table 4, and a representative mandibular reconstruction is shown in Figure 3.

STA, superior thyroid artery; FA, facial artery. Anatomical correlation was considered present when the discrepancy between the preoperative mark and the intraoperative perforator emergence point was ≤1 cm.

All free flaps survived. One patient (10%) developed an intraoral wound dehiscence, which was treated conservatively. No total flap loss, partial skin-paddle necrosis, donor-site ischemia, or postoperative vascular complication was observed. No patient required flap revision because of inadequate perforator perfusion, and there was no need for emergency modification of the skin paddle related to a discrepancy between preoperative ultrasound and intraoperative anatomy.

## 4. Discussion

This study describes a structured surgeon-performed CHFUS protocol specifically adapted to the fibula osteoseptocutaneous flap and demonstrates that the resulting vascular map can be translated with high spatial fidelity into flap elevation. In all 10 patients, the dominant perforator selected preoperatively was found in the expected location, the fascial emergence point was within the predefined 1 cm threshold, and no skin paddle required redesign because of unexpected perforator anatomy. The observed proportion was 100%, but the exact 95% confidence interval was 69.2–100%, which reflects the uncertainty inherent to a 10-patient series. These findings support the feasibility of the protocol and the accuracy of dominant-perforator localization in this cohort, but they should not be interpreted as establishing reliability or reproducibility. Our data do not support replacing CTA with ultrasound for fibula flap planning. They do, however, show that CHFUS provides information that is highly relevant at the time of harvest, especially for perforator selection and individualized skin-paddle design, whereas CTA remains better suited to panoramic evaluation of the donor-limb vasculature.

Fibula flap planning requires a broader vascular assessment than most soft-tissue perforator flaps, because the peroneal artery itself is harvested with the flap. Preoperative imaging must therefore establish both the suitability of the donor limb and the location of the cutaneous perforators supplying the skin paddle. A panoramic vascular study alone may ensure donor-limb safety without providing the level of detail needed for skin-paddle design, while perforator mapping alone is not sufficient if arterial dominance is not assessed. Our protocol addresses these two requirements sequentially within the same examination.

The first phase of the examination uses the same high-resolution linear transducer at a lower operating frequency to gain sufficient penetration for the popliteal artery, tibioperoneal trunk, and proximal peroneal system. This is not intended to reproduce the panoramic three-dimensional dataset of CTA, and the present study was not designed to test ultrasound as a stand-alone exclusion test for every clinically relevant vascular variant. Rather, it demonstrates that a microsurgeon can obtain a coherent real-time view of the arterial anatomy that matters for the subsequent flap design. In our series, the recorded distribution was predominantly Kim–Lippert type I, and no unsafe vascular configuration was identified among patients proceeding to harvest. Because all patients had already been selected for fibula harvest after CTA, these findings reinforce feasibility but cannot establish the sensitivity of CHFUS for rare high-risk variants.

The second phase is where ultrasound becomes particularly relevant to the actual dissection. The cutaneous component of the fibula flap is supplied by branches of the peroneal system whose location and route are variable [7]. A handheld Doppler may indicate that a vessel is present beneath a particular point of skin, but it cannot reliably show whether the signal represents the desired perforator, how large it is, whether it is septocutaneous or musculocutaneous, or where it penetrates the deep fascia [8]. CHFUS provides this anatomical context in real time. The surgeon can follow the vessel from the parent system toward the superficial tissues, rotate the probe to confirm continuity, assess its relationship to the septum and surrounding muscle, and compare alternative perforators before committing to an incision. This is a qualitative difference from acoustic localization alone.

Deep fascial emergence was chosen as the principal surface landmark because it is both sonographically identifiable and surgically reproducible. Mapping a perforator only at its subcutaneous or skin projection can be misleading when the vessel may course obliquely after penetrating the fascia. By marking the fascial crossing point, the surgeon targets the level at which the perforator becomes relevant during flap elevation. Agreement within the predefined ≤10 mm threshold in all 10 patients supports the feasibility of using this landmark for preoperative mapping, but does not establish the precision or reproducibility of the technique.

Patient positioning may further improve correspondence. Ultrasound was performed with the leg positioned as for harvest, whereas CTA is generally acquired in a standardized radiological position. Soft-tissue rotation can shift the apparent surface projection of perforators in the lower limb, and this has been proposed as a source of localization error in CTA-based mapping [18]. Real-time ultrasound avoids this problem by mapping the vessel directly on the positioned limb immediately before surgery.

The findings also fit within the broader evolution of ultrasound in microsurgery. Color duplex imaging was initially used as a more informative alternative to handheld Doppler, but modern high-frequency systems now permit detailed analysis of vessel caliber, fascial planes, and intramuscular course [8,11,12,13,14]. Surgeon-performed protocols have been developed for the SCIP flap and other perforator flaps, demonstrating that the practical value of ultrasound increases when the examination is standardized and performed by the person who will execute the dissection [13,14]. This surgeon-imager model is especially attractive in microsurgery because the relevant question is rarely simply whether a vessel exists. The surgeon wants to know which vessel should be used, how it will be encountered, which tissue planes it traverses, and whether the map requires a change in incision or flap design.

Operator dependence remains an important limitation of surgeon-performed CHFUS. Reproducibility is influenced by probe orientation and handling, transducer pressure, limb position, tissue depth, focal-zone placement, gain, pulse-repetition frequency/velocity scale, wall-filter settings, operating frequency, and platform-specific image processing. These factors become particularly relevant when vessels of approximately 1 mm are being assessed, because small changes in compression or acquisition settings may alter apparent caliber or reduce the visibility of low-flow signals. More broadly, contemporary ultrasound literature has emphasized that probe pressure, positioning, measurement depth, acquisition protocol, and device-related variability can materially affect quantitative ultrasound measurements and should be standardized when reproducibility is an endpoint [20]. In the present protocol, patient position, scanning sequence, preset ranges, low-flow settings, minimal probe pressure, and the level at which perforator caliber was measured were standardized. This improves internal consistency, but the single-operator design does not address interoperator reproducibility and should not be interpreted as evidence that the same performance will be obtained without dedicated training.

The mean peroneal artery diameter at its origin was 2.83 mm, while the dominant perforator measured 1.04 mm at deep fascial emergence. Although these measurements provide additional information on vascular anatomy, perforator selection was not based on caliber alone. Location, course, fascial emergence, and ease of surgical incorporation into the planned skin paddle were considered together during flap design.

CTA provides a rapid panoramic survey of the arterial tree, depicts calcification and occlusive disease, identifies major branching variants, and integrates naturally with the CT-based datasets already used for virtual surgical planning [9,10,21,22]. In a traumatic leg, a limb with previous surgery, suspected peripheral vascular disease, extensive scarring, or an equivocal clinical examination, the global anatomical information of CTA is difficult to replace. Furthermore, because the peroneal artery is sacrificed in a fibula flap, the tolerance for a missed high-risk arterial variant must be extremely low. Our data do not justify removing CTA from the preoperative pathway on the basis of ultrasound alone.

At the same time, CTA is not optimized for every decision the microsurgeon makes when designing the skin paddle. It is static, requires post-processing and interpretation away from the patient, and does not allow the surgeon to interrogate a perforator dynamically during the examination. Ultrasound provides a different set of information that is directly relevant to flap harvest. It allows precise identification of the fascial emergence point, characterization of the perforator as septocutaneous or musculocutaneous, assessment of its course with the limb positioned for surgery, and evaluation of whether the required skin paddle can be reliably centered on the selected vessel without interfering with the planned osseous segment. These findings are primarily operative rather than purely diagnostic. At this stage, ultrasound and CTA should therefore be regarded as complementary modalities, each contributing different information to preoperative planning.

This complementary model is also consistent with comparative literature. González Martínez et al. directly compared CTA, color Doppler ultrasonography, and handheld Doppler in free-flap planning and highlighted the distinct information provided by each modality [10]. Feng and colleagues reported strong performance of color Doppler ultrasound in lower-extremity perforator localization [18], while a systematic review and meta-analysis of ALT perforator imaging found high sensitivity for color Doppler relative to CTA [19]. These data cannot be transferred uncritically to fibula donor-site safety because the consequences of sacrificing the peroneal artery are different, but they support the reliability of ultrasound for the perforator-specific component of planning. The present study extends that concept to the fibula osteoseptocutaneous flap by placing perforator mapping within a broader arterial examination. Preoperative knowledge of the dominant perforator and fascial emergence may allow the skin paddle to be positioned more deliberately in relation to the planned fibular segments and inset, adding soft-tissue vascular information to an otherwise osseous plan.

The absence of major flap-related complications in this small series should not be overinterpreted as an efficacy endpoint. The study was not designed to evaluate flap survival or donor-site morbidity; more relevant to the study question is that no patient required revision for inadequate perforator perfusion and no flap design had to be altered because the preoperative map failed. These process outcomes suggest that CHFUS reduced uncertainty at the point of flap elevation. For an experienced team, this may translate into a more deliberate incision, less exploratory dissection, and greater confidence when choosing a dominant perforator.

The present study has several limitations. It is retrospective, single-center, and includes only 10 patients. Although all 10 selected dominant perforators met the predefined localization criteria, the exact 95% confidence interval for this proportion is 69.2–100%; therefore, the observed 100% agreement is not sufficient to establish reliability or reproducibility. All CHFUS examinations were performed by one reconstructive surgeon. This improves technical consistency but prevents assessment of interoperator performance across different levels of ultrasound experience. In addition, CTA was part of the institutional pathway for every patient, and patients with CTA findings considered unsafe for fibula harvest were excluded before surgery. The study therefore cannot determine whether CHFUS alone would identify all clinically important arterial variants and cannot estimate sensitivity, specificity, or negative predictive value for donor-limb safety. A further limitation is that formal intraoperative localization-error analysis was performed for the selected dominant perforator only. Accordingly, the present data should not be interpreted as a systematic intraoperative validation of all 18 mapped perforators. Moreover, the study focused on anatomical correlation rather than patient-centered or efficiency outcomes. We did not prospectively measure scan duration, flap-harvest time, perforator dissection time, radiological resource use, or cost. Previous reports suggest that high-frequency ultrasound can improve efficiency in thin perforator flap surgery [17], but this should be evaluated specifically for fibula harvest. Future prospective studies should record the time required for the two-phase examination, the frequency with which ultrasound changes donor-leg selection or skin-paddle design, the learning curve among microsurgeons, and whether the protocol reduces exploratory dissection or operative time without compromising safety.

Despite these limitations, the study provides a clear practical message. The value of surgeon-performed CHFUS in fibula flap surgery is not simply finding perforators. Its value lies in linking three levels of information that are usually considered separately: the major arterial configuration of the donor limb, the detailed anatomy of the cutaneous perforator, and the final position of the skin paddle on the patient.

## 5. Conclusions

In this small, retrospective, single-center, single-operator feasibility series, a standardized surgeon-performed CHFUS examination was clinically applicable for preoperative planning of the fibula osteoseptocutaneous free flap. The selected dominant perforator met the predefined ≤1 cm localization threshold in all 10 patients, and the preoperative map could be translated into the planned skin-paddle design without intraoperative redesign related to the selected perforator. The two-phase ultrasound workflow provided a practical sequence from assessment of the main infrapopliteal and peroneal vascular anatomy to high-resolution characterization of the cutaneous perforator and direct skin marking.

The present findings support CHFUS as a complementary operative planning tool rather than a substitute for CTA. CTA remains important for panoramic donor-limb arterial assessment, particularly because the peroneal artery is sacrificed during fibula harvest, whereas CHFUS provides real-time information on perforator caliber, course, deep fascial emergence, and its relationship to the proposed skin paddle. Because all patients in this series were selected for harvest after CTA, this study does not establish CHFUS as a stand-alone screening method for donor-limb vascular safety.

Larger prospective and multicenter studies involving multiple operators are required to define interoperator reproducibility, the learning curve, diagnostic performance for clinically important arterial variants, and any future role of CHFUS as an alternative to CTA in selected patients.

## Figures and Tables

**Table 1:** Recommended ultrasound presets for evaluation of the fibula osteoseptocutaneous flap.

**B-mode** (Morphological assessment of the infrapopliteal arterial axes, peroneal artery, and cutaneous perforators.)	Linear
Probe selection	
Frequency (Frq)	10–20 MHz
Gain (Gn)	26–34 dB
Depth (D)	2.2–2.9 cm
Focus	1–2 cm
**Color flow (CF)** (Flow assessment and tracking of the peroneal artery and its cutaneous perforators)	Minimum
Wall Filter (WF)	
Gain (Gn)	8–18 dB
Frequency (Frq)	12–15 MHz
PRF/Velocity Scale	0.7–1.3 kHz/4–9.5 cm/s

**Table 2:** Patient demographics and osseous defect site.

Patient	Sex	Age (Years)	BMI (kg/m^2^)	Osseous Defect Site
1	F	53	19.91	Mandible
2	M	68	27.81	Maxilla
3	M	59	22.32	Mandible
4	F	55	22.11	Maxilla
5	M	19	19.51	Mandible
6	F	65	20.76	Mandible
7	M	38	26.02	Maxilla
8	F	52	23.68	Mandible
9	M	75	24.42	Mandible
10	M	37	23.21	Mandible
Mean ± SD	60% M/40% F	52.10 ± 16.69	22.98 ± 2.64	70% mandible/30% maxilla

M, male; F, female; SD, standard deviation; BMI, body mass index.

**Table 3:** Preoperative CHFUS findings and perforator characteristics.

Patient	Kim–Lippert Type	Number of Mapped Perforators (*n*)	d1 (mm)	d2 (mm)	D1 (cm)	D2 (cm)	D3 (cm)	Course of All Mapped Perforators
1	I	2	2.61	0.92	10.8	17.9	-	P1: [SC]; P2: [SC] Dominant: P2
2	I	1	2.94	1.08	12.4	-	-	SC
3	I	2	2.78	1.17	13.1	21.8	-	P1: [SC]; P2: [SC] Dominant: P2
4	I	2	2.85	0.97	11.2	18.5	-	P1: [SC]; P2: [MC] Dominant: P1
5	I	1	2.67	0.84	12.0	-	-	SC
6	IIA	3	3.11	1.26	10.9	17.6	22.4	P1: [SC]; P2: [SC]; P3: [SC] Dominant: P3
7	I	2	2.88	1.12	13.4	21.5	-	P1: [SC]; P2: [MC] Dominant: P2
8	I	1	2.73	0.89	11.8	-	-	SC
9	I	2	2.96	1.03	10.7	18.2	-	P1: [SC]; P2: [SC] Dominant: P1
10	I	2	2.81	1.15	12.6	20.9	-	P1: [SC]; P2: [MC] Dominant: P1
Summary	-	1.80 ± 0.63	2.83 ± 0.15	1.04 ± 0.14	11.89 ± 0.98	19.49 ± 1.89	-	SC: 15/18 MC: 3/18

d1, ultrasound diameter of the peroneal artery at its origin from the tibioperoneal trunk; d2, ultrasound diameter of the dominant perforator at deep fascial emergence; D1–D3, distance from the fibular head to the first, second, and third mapped perforators; SD, standard deviation; P1–P3, respectively Perforator 1, Perforator 2 and Perforator 3; SC, septocutaneous; MC, musculocutaneous.

**Table 4:** Intraoperative characteristics and outcomes.

Patient	Recipient Artery	Skin-Paddle Dimensions (cm)	Localization Distance (mm)	Anatomical Agreement ≤ 1 cm	Perforators Included (n)	Flap or Donor-Site Complications	Anatomical Inset Site
1	STA	6 × 8	1.2	Yes	1	Intraoral wound dehiscence	Mandible
2	FA	8 × 8	2.4	Yes	1	-	Maxilla
3	STA	6 × 8	3.1	Yes	1	-	Mandible
4	FA	6 × 8	4.0	Yes	1	-	Maxilla
5	STA	7 × 7	4.8	Yes	1	-	Mandible
6	STA	8 × 7	5.6	Yes	1	-	Mandible
7	STA	6 × 7	6.3	Yes	1	-	Maxilla
8	STA	8 × 8	7.2	Yes	1	-	Mandible
9	STA	7 × 8	8.5	Yes	1	-	Mandible
10	STA	6 × 6	9.7	Yes	1	-	Mandible

STA, superior thyroid artery; FA, facial artery; SD, standard deviation; CI, confidence interval. Localization distance was defined as the distance between the preoperative ultrasound skin marking of the selected dominant perforator and its corresponding intraoperative deep fascial emergence point. Anatomical agreement was defined as a localization distance of ≤1 cm and was observed in 10/10 patients (100%; exact two-sided 95% CI calculated using the Clopper-Pearson method, 69.2–100%).

## Data Availability

The data supporting the findings of this study are available from the corresponding author upon reasonable request. The data are not publicly available due to patient privacy considerations.

## Supplementary Materials

The following supporting information can be downloaded at: https://www.mdpi.com/article/10.3390/jcm15207768/s1, Video S1: Color-Mode Transverse CHFUS of the Peroneal Vessels and their perforators.
